# Antimicrobial susceptibility profiles of *Mycoplasma hyorhinis* strains isolated from five European countries between 2019 and 2021

**DOI:** 10.1371/journal.pone.0272903

**Published:** 2022-08-11

**Authors:** Ulrich Klein, Dorottya Földi, Nikolett Belecz, Veronika Hrivnák, Zoltán Somogyi, Michele Gastaldelli, Marianna Merenda, Salvatore Catania, Arkadiusz Dors, Ute Siesenop, Philip Vyt, Zsuzsa Kreizinger, Wouter Depondt, Miklós Gyuranecz

**Affiliations:** 1 Huvepharma NV, Antwerp, Belgium; 2 Veterinary Medical Research Institute, Budapest, Hungary; 3 University of Veterinary Medicine, Budapest, Hungary; 4 Istituto Zooprofilattico Sperimentale della Venezie, Verona, Italy; 5 National Veterinary Research Institute, Puławy, Poland; 6 University of Veterinary Medicine Hanover, Hanover, Germany; 7 Dialab Diagnostic Laboratory, Belsele, Belgium; 8 MolliScience Kft., Biatorbágy, Hungary; Academia Sinica, TAIWAN

## Abstract

*Mycoplasma hyorhinis* is an emerging swine pathogen bacterium causing polyserositis and polyarthritis in weaners and finishers. The pathogen is distributed world-wide, generating significant economic losses. No commercially available vaccine is available in Europe. Therefore, besides improving the housing conditions for prevention, antimicrobial therapy of the diseased animals is the only option to control the infection. Our aim was to determine the minimal inhibitory concentrations (MIC) of ten antimicrobials potentially used against *M*. *hyorhinis* infection. The antibiotic susceptibility of 76 *M*. *hyorhinis* isolates from Belgium, Germany, Hungary, Italy and Poland collected between 2019 and 2021 was determined by broth micro-dilution method and mismatch amplification mutation assay (MAMA). Low concentrations of tiamulin (MIC_90_ 0.312 μg/ml), doxycycline (MIC_90_ 0.078 μg/ml), oxytetracycline (MIC_90_ 0.25 μg/ml), florfenicol (MIC_90_ 2 μg/ml) and moderate concentrations of enrofloxacin (MIC_90_ 1.25 μg/ml) inhibited the growth of the isolates. For the tested macrolides and lincomycin, a bimodal MIC pattern was observed (MIC_90_ >64 μg/ml for lincomycin, tulathromycin, tylosin and tilmicosin and 5 μg/ml for tylvalosin). The results of the MAMA assay were in line with the conventional method with three exceptions. Based on our statistical analyses, significant differences in MIC values of tiamulin and doxycycline were observed between certain countries. Our results show various levels of antimicrobial susceptibility among *M*. *hyorhinis* isolates to the tested antibiotics. The data underline the importance of susceptibility monitoring on pan-European level and provides essential information for proper antibiotic choice in therapy.

## Introduction

*Mycoplasma hyorhinis*, a member of the Mollicutes class, is a swine facultative pathogen bacterium present worldwide. *M*. *hyorhinis* is commonly found in the ciliated upper respiratory tract of swine, can be shed through nasal secretion or oral fluids and transmitted through direct contact of sows and pigs [1–3]. Clinical signs, which are most frequently polyserositis and polyarthritis can appear in three to ten week old weaners and finishers. Infrequently, *M*. *hyorhinis* is also associated with conjunctivitis, otitis media, pneumonia and, as a secondary pathogen, aggravates the symptoms of porcine reproductive and respiratory syndrome and enzootic pneumonia [4].

As of today, no commercial vaccine against *M*. *hyorhinis* infection is available in Europe. The control of the infection therefore mainly relies on improving housing conditions, protection against other pathogens and antibiotic treatment. Members of the Mollicutes class are cell-wall-less organisms, therefore intrinsically resistant to β-lactam antibiotics. Members of this class are also resistant to the RNA polymerase inhibiting rifamycins [5]. Generally, *Mycoplasma* species are sensitive to protein and nucleic acid synthesis inhibiting antibiotics, like pleuromutilins, tetracyclines, fluoroquinolones, aminoglycosides, amphenicols, macrolides and lincosamides [6]. However, *M*. *hyorhinis* is also intrinsically resistant to 14-membered macrolides [7].

Resistance against macrolide and lincosamide antimicrobial agents was previously reported from Asia [7, 8] and Hungary [9]. A single nucleotide polymorphism (SNP) at position A2066G (numbering based on the 23S rRNA gene of the *M*. *hyorhinis* type strain NCTC10130) has been identified as a molecular marker for macrolide and lincomycin resistance [7, 10]. A mismatch amplification mutation assay (MAMA) was developed to detect this SNP, which provides a convenient and cost-effective method to characterize macrolide and lincomycin susceptibility of the isolates. With the MAMA assay, genotypes associated with high (H) or low (L) minimal inhibitory concentration (MIC) values can be distinguished based on the melting temperatures of the amplicons in a real-time PCR platform [10].

The aim of the study was to determine antibiotic susceptibility profiles of *M*. *hyorhinis* isolates recovered from five different European countries against ten antibiotics licensed for veterinary use and approved for medication in pigs by broth micro-dilution method and, where applicable, with molecular biological tools.

## Materials and methods

In total 76 *M*. *hyorhinis* isolates collected between 2019 and 2021 from affected swine showing clinical signs of *M*. *hyorhinis* infection were tested in this study (S1 Table). The isolates originated from farms in Belgium (n = 2), Germany (n = 15), Hungary (n = 20), Italy (n = 20) and Poland (n = 19). Filter-cloned isolates were transferred to the central laboratory for antimicrobial susceptibility testing, where the viability and purity of the isolates were checked before testing. Presence of other *Mycoplasma* species in the cultures was excluded by a set of polymerase chain reactions (PCR) preceded by DNA extraction. DNA extraction was performed by ReliaPrep gDNA Tissue Miniprep System (Promega Inc., Madison, USA) according to the manufacturers’ instructions for animal tissue samples. All isolates were identified by *M*. *hyorhinis* specific real-time PCR [11] and presence of other swine mycoplasma species was excluded by either real-time PCR for *M*. *hyopneumoniae* [11] and *M*. *hyosynoviae* [12] or conventional PCR for *M*. *flocculare* [13]. To detect the presence of other possible contaminant *Mycoplasma* species, a universal *Mycoplasma* sp. specific PCR was also performed targeting the intergenic spacer region of 16S rRNA and 23S rRNA genes [14]. Only pure *M*. *hyorhinis* cultures were included in the antibiotic susceptibility testing.

Throughout the tests, Mycoplasma Liquid Media (Mycoplasma Experience Ltd., Bletchingley, UK) was used as culture medium. The number of colour changing units (CCU) were calculated by plate micro-dilution from the highest dilution showing colour change (red to yellow shift) [15].

The following antimicrobials were tested: one pleuromutilin (tiamulin), two tetracyclines (doxycycline, oxytetracycline), one phenicol (florfenicol), one fluoroquinolone (enrofloxacin), four macrolides (tylosin, tilmicosin, tylvalosin, tulathromycin), and one lincosamide (lincomycin). Tylvalosin originated from ECO^®^ Animal Health Ltd., UK, tulathromycin originated from Pfizer^®^ Inc., USA and the rest of the compounds originated from Vetranal^®^, Sigma-Aldrich, USA. Stock solutions in the concentration of 1 mg/ml were prepared, aliquoted and stored frozen at -70°C until use. Twofold dilutions were freshly prepared before the tests in the range of 0.039–10 μg/ml for tiamulin, tylvalosin, doxycycline and enrofloxacin, 0.125–32 μg/ml for oxytetracycline and florfenicol and 0.25–64 μg/ml for tylosin, tilmicosin, tulathromycin and lincomycin. The broth micro-dilution test was performed in 96-well microtiter plate containing twofold dilution series of the antibiotic, with sterility, pH and growth control. The clinical isolates were tested in duplicates and each day, the *M*. *hyorhinis* type strain (NCTC 10130) was included in the tests as quality control. All isolates were tested at the viable count of 10^5^ CCU/ml. The MIC value of each isolate was defined as the lowest concentration of the antibiotic where no colour change (no growth) was recorded by the time the growth control changed colour [15]. MIC_50_ and MIC_90_ values were defined as the lowest concentrations that inhibited the growth of 50% and 90% of the tested isolates [15].

The statistical analyses presented in this work were conducted under R environment [16]. First, an extended Cochran Armitage test was performed with a null hypothesis proposing that the distribution of the frequency of each MIC value was independent of the country (package “coin”) [17]. Since multiple antibiotics were considered at the same time, p-values were adjusted for multiple comparisons by Benjamini-Hochberg method. In the cases where significant differences were found a proportional odds model for each antibiotic was constructed relating the cumulative frequency distribution of each MIC value to the variable country. The models were constructed with the function *clm* of the package “ordinal” [18], implemented a logit link function and assumed equidistant thresholds (for model parameter estimation, see S2 Table). The validity of the proportional odds assumption was assessed with the function *nominal* of the same package. Pairwise estimate comparison among countries were carried out with the package “emmeans” (https://github.com/rvlenth/emmeans). Estimates are presented in the logit form.

The mismatch amplification mutation assay was performed based on the primer sequences and conditions described earlier [10] using a Bio-Rad C1000 Touch^™^ Thermal Cycler, CFX96^™^ Real-Time System (Bio-Rad Laboratories Inc., USA). The 23S rRNA sequencing primers Mhr-D5-1F and Mhr-D5-1R published earlier [7] were used to amplify a 192 bp long product which was sequenced on an ABI Prism 3100 automated DNA sequencer (Applied Biosystems, USA). The PCR master mix consisted of 25 μl 2× PCRBIO HS Taq Mix Red (PCR Biosystems Ltd., UK), 4 μl of each primer (10 μM) and 4 μl DNA in the final volume of 50 μl. PCR conditions were the following 95°C for one minute, 40 cycles of 95°C for 15 seconds, 60°C for 15 seconds and 72°C for 40 seconds.

## Results

**Fig 1:**
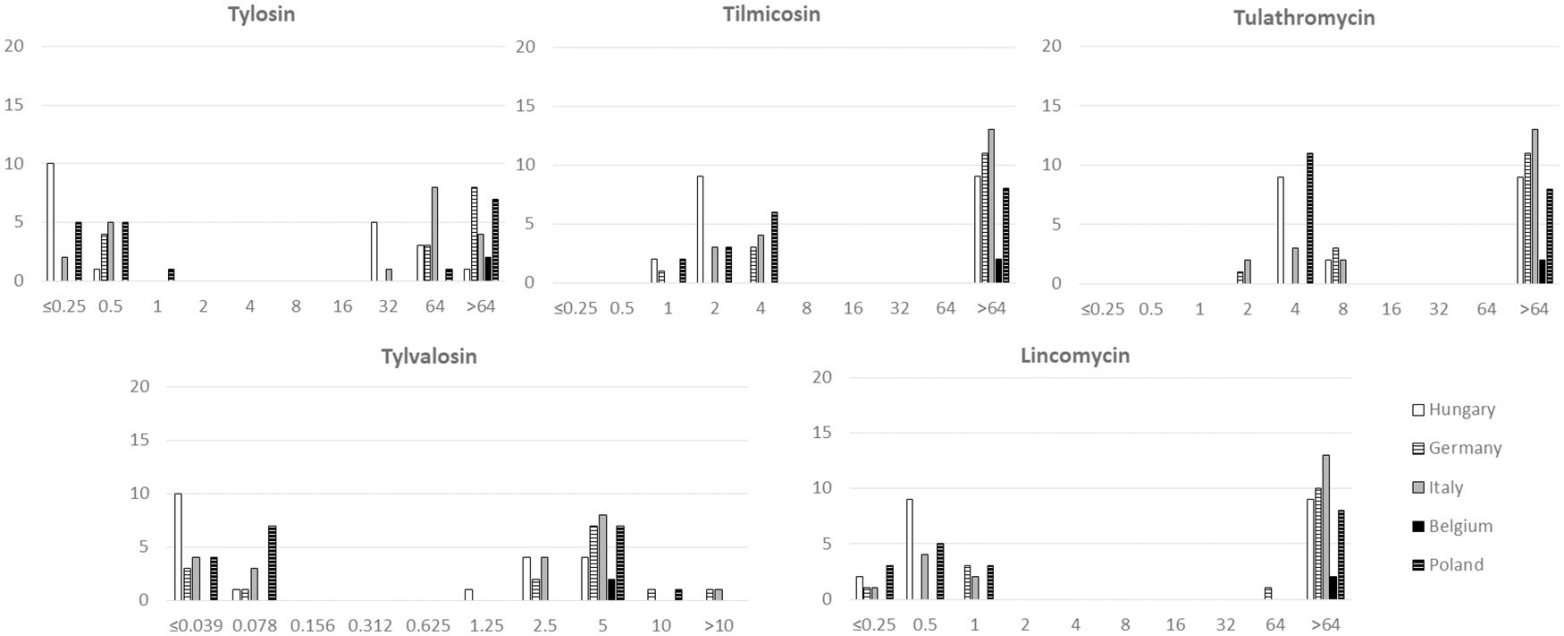
Minimal inhibitory concentration (MIC) distribution of the 76 tested *Mycoplasma hyorhinis* isolates for macrolides (tylosin, tilmicosin, tylvalosin, tulathromycin) and lincomycin by country of origin. X-axis: concentration of the different antimicrobials (μg/ml); Y-axis: number of isolates.

**Fig 2:**
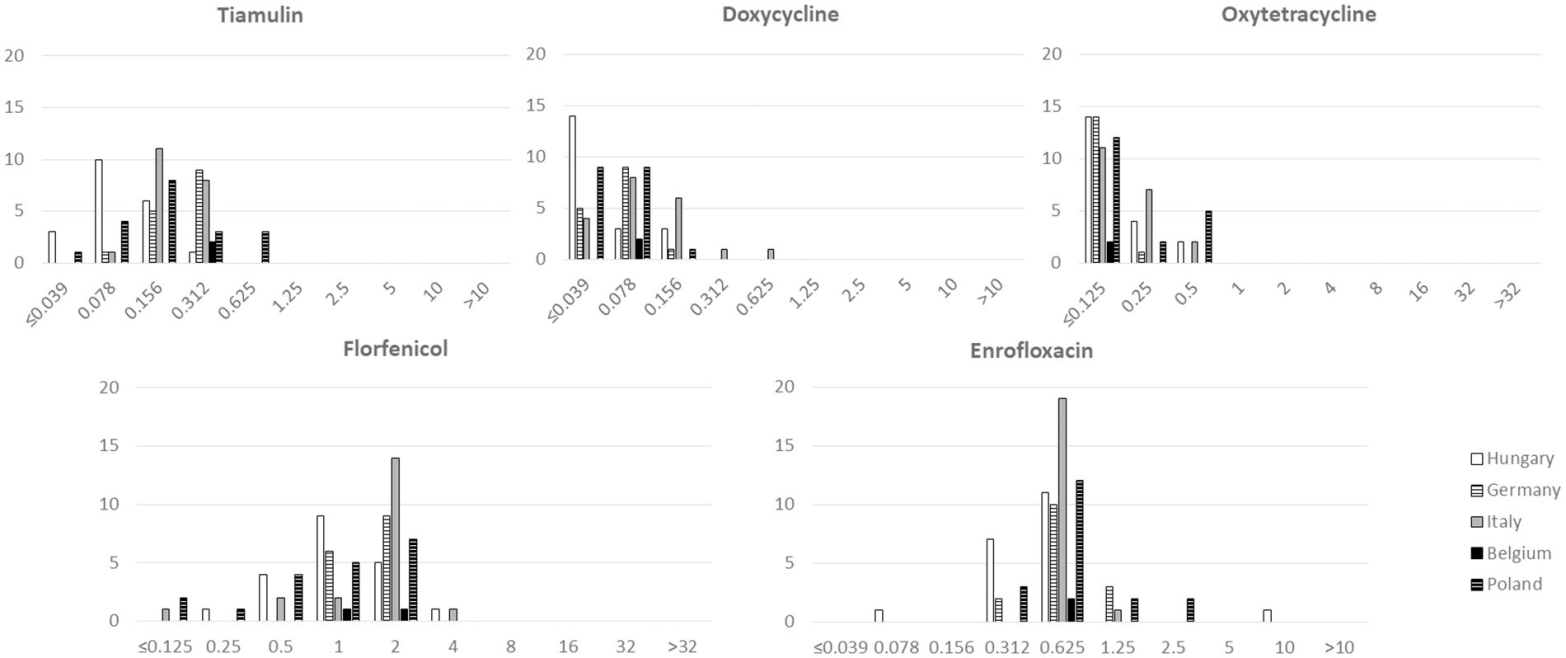
Minimal inhibitory concentration (MIC) distribution of the 76 tested *Mycoplasma hyorhinis* isolates for tiamulin, doxycycline, oxytetracycline, florfenicol and enrofloxacin by country of origin. X-axis: concentration of the different antimicrobials (μg/ml); Y-axis: number of isolates.

The determined MIC parameters (MIC range, MIC_50_ and MIC_90_) are listed separately by individual countries and for all tested isolates in Table 1. Detailed MIC results of the tested antibiotic agents are described in S1 Table. Regardless of the country of origin, two different susceptibility patterns were determined: a bimodal MIC distribution for macrolides and lincomycin (Fig 1) and a unimodal distribution for the other tested antimicrobial agents (Fig 2). The tested isolates were inhibited by low concentrations of tiamulin (MIC_90_ 0.312 μg/ml), doxycycline (MIC_90_ 0.078 μg/ml), oxytetracycline (MIC_90_ 0.25 μg/ml) and florfenicol (MIC_90_ 0.2 μg/ml). Moderate concentrations of enrofloxacin (MIC_90_ 1.25 μg/ml) inhibited the growth of most of the isolates with one exception against a Hungarian isolate (Hu-20) where 10 μg/ml (the highest concentration tested) was detected. In 43 cases out of the 76 isolates (56% of the isolates), high MICs were detected for all the tested macrolide agents (tylosin, tilmicosin, tylvalosin and tulathromycin) and lincomycin.

**Table 1:** Minimal inhibitory concentration (MIC) values of ten antimicrobial agents against 76 *Mycoplasma hyorhinis* isolates. Total values and values for each country are given.

Country of Origin	MIC parameter	Tia	Dox	Oxy	Flo	Enr	Tyl	Til	Tyv	Tul	Lin
Belgium (2 isolates)	MIC	0.312	0.078	≤0.125	1–2	0.625	>64	>64	5	>64	>64
Germany (15 isolates)	MIC Range	0.078–0.312	≤0.039–0.156	≤0.125–0.25	1–2	0.312–1.25	0.5 –>64	1 –>64	≤0.039 –>10	2 –>64	≤0.25 –>64
	MIC_50_	0.312	0.078	≤0.125	2	0.625	>64	>64	5	>64	>64
	MIC_90_	0.312	0.078	≤0.125	2	1.25	>64	>64	10	>64	>64
Hungary (20 isolates)	MIC Range	≤0.039–0.312	≤0.039–0.156	≤0.125–0.5	0.25–4	0.078–10	≤0.25 –>64	1 –>64	≤0.039–5	4 –>64	≤0.25 –>64
	MIC_50_	0.078	≤0.039	≤0.125	1	0.625	≤0.25	2	≤0.039	8	0.5
	MIC_90_	0.156	0.156	0.25	2	0.625	64	>64	5	>64	>64
Italy (20 isolates)	MIC Range	0.078–0.312	≤0.039–0.625	≤0.125–0.5	≤0.125–4	0.625–1.25	≤0.25 –>64	2 –>64	≤0.039 –>10	2 –>64	≤0.25 –>64
	MIC_50_	0.156	0.078	≤0.125	2	0.625	64	>64	2.5	>64	>64
	MIC_90_	0.312	0.156	0.25	2	0.625	>64	>64	5	>64	>64
Poland (19 isolates)	MIC Range	≤0.039–0.625	≤0.039–0.156	≤0.125–0.5	≤0.125–2	0.312–2.5	≤0.25 –>64	1 –>64	≤0.039–10	4 –>64	≤0.25 –>64
	MIC_50_	0.156	≤0.039	≤0.125	1	0.625	0.5	4	0.078	4	1
	MIC_90_	0.625	0.078	0.5	2	1.25	>64	>64	5	>64	>64
All 76 isolates	MIC Range	≤0.039–0.625	≤0.039–0.625	≤0.125–0.5	≤0.125–4	≤0.039–10	≤0.25 –>64	1 –>64	≤0.039 –>10	2 –>64	≤0.25 –>64
	MIC_50_	0.156	0.078	≤0.125	1	0.625	32	>64	2.5	>64	>64
	MIC_90_	0.312	0.078	0.25	2	1.25	>64	>64	5	>64	>64

Abbreviations: Tia = tiamulin, Dox = doxycycline, Oxy = oxytetracycline, Flo = florfenicol, Enr = enrofloxacin, Tyl = tylosin, Til = tilmicosin, Tyv = tylvalosin, Tul = tulathromycin, Lin = lincomycin.

Considering the quantity of isolates available from each country, statistical analyses were performed with isolates from Germany (n = 15), Hungary (n = 20), Italy (n = 20) and Poland (n = 19). Results of the extended Cochran Armitage test showed significant differences in MIC value frequencies between countries in the case of tiamulin and doxycycline (Table 2). Further analyses of tiamulin MIC profiles indicate that isolates from Hungary display the lowest MIC profiles, while the other three countries present similar MIC profiles with higher tiamulin MIC values (Table 3). For doxycycline, significant differences in MIC profiles were detected between Hungary and Italy and Poland and Italy, with isolates from Italy representing higher doxycycline MIC values (Table 3).

**Table 2:** Extended Cochran-Armitage test of minimal inhibitory concentration class frequency versus country.

Antibiotic	X^2^	p-value	Adjusted p-value
**Tiamulin**	**20.61**	**<0.05**	**<0.05**
**Doxycycline**	**13.17**	**<0.05**	**<0.05**
Oxytetracycline	6.17	0.10	0.14
Florfenicol	8.08	0.04	0.10
Enrofloxacin	4.45	0.22	0.28
Tylosin	10.01	0.02	0.06
Tilmicosin	7.84	0.05	0.10
Tylvalosin	6.69	0.08	0.14
Tulathromycin	5.65	0.13	0.15
Lincomycin	5.93	0.12	0.14

Significant differences are highlighted by bold lettering.

**Table 3:** Pairwise estimate comparisons of the proportional odds model relating the frequency of observation of the different classes of tiamulin and doxycycline minimal inhibitory concentrations to the variable country.

Tiamulin				
Contrasts	Estimate	Standard Error	Z-value	adjusted p-value
**Germany—Hungary**	**3.17**	**0.72**	**4.40**	**0.05**
Germany—Italy	0.50	0.61	0.82	0.41
Germany—Poland	1.08	0.68	1.59	0.17
**Hungary—Italy**	**-2.67**	**0.66**	**-4.08**	**<0.05**
**Hungary—Poland**	**-2.10**	**0.68**	**-3.09**	**<0.05**
Italy—Poland	0.58	0.63	0.92	0.41
Doxycycline				
Contrasts	Estimate	Standard Error	Z-value	adjusted p-value
Germany—Hungary	1.10	0.68	1.63	0.16
Germany—Italy	-1.20	0.64	-1.87	0.12
Germany—Poland	0.42	0.63	0.68	0.50
**Hungary—Italy**	**-2.30**	**0.68**	**-3.37**	**<0.05**
Hungary—Poland	-0.68	0.65	-1.04	0.36
**Italy—Poland**	**1.62**	**0.63**	**2.58**	**<0.05**

Significant differences are highlighted by bold lettering.

Results of the MAMA for macrolide and lincomycin susceptibility were in line with the results of the conventional broth micro-dilution test, except for one German (Ge-13) and two Polish (Po-4 and Po-9) isolates. Based on sequencing results of the partial 23S rRNA gene of these isolates (see the alignment in S1 File) at position 2066 (numbered according to 23S rRNA gene of the *M*. *hyorhinis* type strain NCTC 10130; nucleotide 92 in the alignment), an adenine was found in Po-4 and Po-9 and a thymine in Ge-13 instead of the expected guanine in H genotypes (isolates inhibited with only high concentrations of the antibiotics).

## Discussion

The availability of recent and comparable MIC data from the literature is essential for the selection of the antibiotic of choice for the therapy of *M*. *hyorhinis* infections, as *in vitro* antibiotic susceptibility testing of veterinary *Mycoplasma* strains is still not standardised, is time consuming and requires special expertise. Information on susceptibility trends of European *M*. *hyorhinis* isolates is limited and often restricted to specific countries. The current study aimed to reveal the antibiotic susceptibility profiles of recent European *M*. *hyorhinis* isolates collected in European countries with considerable pig production sectors and compare the data with previous results. Low concentrations of tiamulin, doxycycline, oxytetracycline florfenicol and moderate concentrations of enrofloxacin inhibited the growth of the tested isolates. Conversely, a bimodal distribution of MICs was observed in the tested macrolides and lincomycin with 56% of the tested isolates showing low susceptibility to these drugs. In order to detect the changes in susceptibility of *M*. *hyorhinis* isolates from Europe over several years, a comparison between previous publications and our results was performed (Table 4). As there is no official, standardised guideline for the antibiotic susceptibility testing of veterinary *Mycoplasma* species, only studies which applied a similar antibiotic susceptibility testing method and revealed MIC values of the *M*. *hyorhinis* type strain (NCTC 10130) in the same range as the present study (Table 4) were included in the comparison [9, 19, 20]. These studies all applied the recommended guidelines for antibiotic susceptibility testing of veterinary *Mycoplasma* species according to the work of Hannan [15]. Consistent low MIC values and highest *in vitro* activity of tiamulin, doxycycline and oxytetracycline are observed over a time period of around 35 years in the studies listed above and in our work. No trend of MIC increases was determined which gives prominence to these antimicrobials as being most effective against the tested *M*. *hyorhinis* isolates. Based on the comparison of MIC_50_ and MIC_90_ values of our study with the data of the publications, striking divergences in the tylosin and lincomycin MIC values were detected. Accordingly, isolates exhibiting high MIC values of these antibiotics appeared after 2010, described by Bekő and co-workers [9] and in our study. This published information is useful in assessing corresponding changes in the sensitivity pattern of *M*. *hyorhinis* isolates in different European countries over longer time periods.

**Table 4:** Comparison of minimal inhibitory concentrations (MIC) of ten antimicrobial agents from previous publications and the present study. MIC values against *Mycoplasma hyorhinis* type strain (NCTC 10130 = BTS-7) gained in the different studies are also given.

	The Netherlands, 1984–1989 (n = 20) [19]			USA, Japan and Europe, before 1997 (n = 20) [20]			Hungary, 2014–2017 (n = 38) [9]			Belgium, Germany, Hungary, Italy, Poland 2019–2021 (n = 76) ^a^		
	MIC_50_	MIC_90_	Type strain MIC	MIC_50_	MIC_90_	Type strain MIC	MIC_50_	MIC_90_	Type strain MIC	MIC_50_	MIC_90_	Type strain MIC
**Tia**	0.06	0.12	≤0.03	0.1	0.25	0.05	0.156	0.312	0.078	0.156	0.312	0.078
**Dox**	≤0.03	0.12	≤0.03				0.078	0.312	≤0.039	0.078	0.078	≤0.039
**Oxy**	0.12	0.25	0.12	0.25	2.5	0.05	≤0.25	1	≤0.25	≤0.125	0.25	≤0.125
**Flo**							2	2	1	1	2	2
**Enr**				0.5	1	0.5	0.625	0.625	0.625	0.625	1.25	0.625
**Tyl**	0.12	0.25	0.06	0.5	2.5	0.5	0.5	>64	≤0.25	32	>64	≤0.25
**Til**							2	>64	1	>64	>64	2
**Tyv**							≤0.25	8	≤0.25*	2.5	5	≤0.039*
**Tul**							4	>64	2	>64	>64	4
**Lin**	0.5	1	0.5				0.5	>64	0.5	>64	>64	0.5

^a^ present study.

Abbreviations: Tia = tiamulin, Dox = doxycycline, Oxy = oxytetracycline, Flo = florfenicol, Enr = enrofloxacin, Tyl = tylosin, Til = tilmicosin, Tyv = tylvalosin, Tul = tulathromycin, Lin = lincomycin.

*Due to changing the tested concentration range for tylvalosin, the minimal tested concentration changed from 0.25 mg/ml to 0.039 mg/ml between the two studies.

Our study permits some comparisons between the participating countries and the provided *M*. *hyorhinis* isolates. Significant differences among the isolates from the different countries were detected as isolates from Hungary presented lower MIC values as opposed to the other isolates for tiamulin and doxycycline. These differences can be explained either by the differences in the route of administration (feed/water or parenteral) of the drugs or by the discrepancies in the recommended treatment dosages between countries as described earlier [21]. Differences in the frequency of use can also be hypothesised amongst different countries.

The macrolide and lincomycin susceptibility of the *M*. *hyorhinis* isolates collected in the present study was also tested by a MAMA [10] and, based on the total number of examined isolates (76 isolates in the present study in addition to the published isolates), the true detection rate of the assay for tylosin, tulathromycin and lincomycin is 123/126 (97.62%) and 122/126 (96.83%) for tilmicosin and tylvalosin. Based on the information of this molecular biological assay in most of the isolates, a single nucleotide polymorphism of the 23S rRNA gene contributes to evade the effect of macrolides and lincomycin, as this SNP (A2066G; numbering according to the *M*. *hyorhinis* type strain NCTC 10130 23S rRNA gene) results in a target site modification [22]. Nonetheless, the sequences of the isolates Ge-13, Po-4 and Po-9 presented here display that other resistance mechanisms are present in this pathogen. Resistance to macrolides can also be the result of methylation of nucleotides in domains II or V of the 23S rRNA gene or efflux mechanisms. However, neither has been described in *Mycoplasma* species yet [22]. The formation of biofilms can also support the survival of bacteria. As described in cases of *M*. *hyopneumoniae*, cells in biofilms were inhibited by significantly higher concentrations of antibiotics than planktonic cells [23]. The pharmacokinetic characteristics of used antimicrobial agents is also crucial for their provided clinical efficacy. In the present study, the tested ten antimicrobials (doxycycline, oxytetracycline, florfenicol, enrofloxacin, tylosin, tilmicosin, tylvalosin, tulathromycin, lincomycin and tiamulin) are characterized by excellent distribution and are able to achieve high concentration in tissues [24–33]. In addition, two of the antimicrobials (florfenicol and tiamulin) have been described as able to achieve therapeutic concentrations in the joint synovial fluid of pigs [34, 35]. These properties of the antimicrobials are crucial for the therapy against a pathogen like *M*. *hyorhinis*, which causes systemic lesions and polyarthritis in infected animals. These factors influence the *in vivo* efficacy of the antibiotics, which should be considered during the evaluation of the results of *in vitro* antibiotic susceptibility tests and molecular biological assays.

In this study, the MIC values of therapeutically important antibiotics were determined and susceptibility trends of European *M*. *hyorhinis* isolates were compared between countries and over years. Based on the molecular biological information provided by the MAMA tests, resistance mechanisms besides target site modification should be considered in case of macrolides and lincomycin.

## Conclusions

Based on the 76 tested European *M*. *hyorhinis* isolates, doxycycline, oxytetracycline and tiamulin proved to be the most effective compounds *in vitro*. Detected bimodal MIC patterns for the tested macrolides and lincomycin and the determined country-specific differences of MIC values for tiamulin and doxycycline among the examined European countries emphasize the importance of susceptibility monitoring on a Pan-European level and strengthens the need for the introduction of standardized MIC testing and the development of breakpoints for MIC result interpretation.

## Supporting information

S1 Table Background information of the tested *Mycoplasma hyorhinis* isolates and results of the broth micro-dilution test and mismatch amplification mutation assay. (XLSX)

S2 Table Parameter estimates with relative standard error, Wald statistic (Z-value) and p-value of the proportional odds model relating the frequency of observation of the different minimal inhibitory concentration values of tiamulin and doxycycline to the variable country. (DOCX)

S1 File Alignment of the partial 23S rRNA gene sequences of the *Mycoplasma hyorhinis* type strain (NCTC 10130) Ge-13, Po-4 and Po-9. (TXT)
