# Supplementary material for: Antimicrobial susceptibility profiles of Mycoplasma hyorhinis strains isolated from five European countries between 2019 and 2021
Source: PLoS One. 2022 Aug 11;17(8):e0272903. doi: 10.1371/journal.pone.0272903 (PMC9371350; doi:10.1371/journal.pone.0272903)
Supplement: S2 Table — (DOCX) [file pone.0272903.s002.docx]

| Tiamulin | | | | |
| --- | --- | --- | --- | --- |
|  | Estimate | Standard Error | Z-value | p-value |
| Coefficients |  |  |  |  |
| Country_Hungary | -3.17 | 0.72 | -4.40 | 1.10⋅10^-5^ |
| Country_Italy | -0.50 | 0.61 | -0.82 | 0.41 |
| Country_Poland | -1.08 | 0.68 | -1.59 | 0.11 |
| Threshold coefficients |  |  |  |  |
| threshold.1 | -4.92 | 0.73 | -6.77 |  |
| spacing | 2.38 | 0.27 | 8.81 |  |
| Doxycycline | | | | |
|  | Estimate | Standard Error | Z-value | p-value |
| Coefficients |  |  |  |  |
| Country_Hungary | -1.10 | 0.68 | -1.63 | 0.10 |
| Country_Italy | 1.20 | 0.64 | 1.87 | 0.06 |
| Country_Poland | -0.42 | 0.63 | -0.68 | 0.50 |
| Threshold coefficients |  |  |  |  |
| threshold.1 | -0.39 | 0.47 | -0.84 |  |
| spacing | 2.04 | 0.30 | 6.88 |  |
